# Calcarea-carbonica

**Published:** 1897-05

**Authors:** 


### HOMŒOPATHIC PHYSICIAN,

A MONTHLY JOURNAL OF

HOMŒOPATHIC MATERIA MEDICA AND CLINICAL MEDICINE.

"If our school ever gives up the strict inductive method of Hahnemann, we are lost, and deserve only to be mentioned as a caricature in the history of medicine."—CONSTANTINE HERING.

Vol. XVII. MAY, 1897. No. 5.

### EDITORIAL.

CALCAREA-CARBONICA.—This remedy is one of the most familiar drugs of the homeopathic materia medica, and one most frequently prescribed; yet, like Aconite, it is not well understood. Some practitioners look upon it as a food, and imagine that when they prescribe it, it will be built up into bone tissue. Hence they give it in the very lowest potencies in conformity with this chemico-physiological notion, and so lose the great benefits to be derived from its potencies, prescribed strictly upon the symptomatology of the provings.

It may be said with confidence that these inorganic elements which form a part of the human economy are not, except in one or two instances, assimilated and built up into the tissues when presented in their normal state to the digestive system. They must be combined with the protein bodies, such, for instance, as the gluten of wheat, before they can be appropriated and form part of the animal economy.

The prescription of Calcarea, therefore, from the chemical point of view, is an absurdity for a homeopathic physician; and its clinical results under such circumstances will prove as much a disappointment as its administration in potency exactly in accordance with the symptoms in the pathogenesis will prove a gratifying surprise and pleasure.

Calcarea is a great remedy in delirium tremens, and hence one of its characteristics is "delirious talk about rats, mice, fire, and murder."

One of its great characteristics is "fear of loss of reason." Cuprum-metallicum has this same symptom.

Calcarea has vertigo when ascending a height, and from looking upwards. This word "ascending" suggests a number of indications for remedies under varying circumstances. Several of them are quite useful, and are as follow: Guernsey's keynotes for Calcarea are "vertigo on running upstairs," and "shortness of breath on going upstairs; must sit down and rest before arriving at the top."

Calcarea-carbonica, vertigo on ascending stairs, with *inclination to fall to the left.*

Iodine, remarkable and unaccountable weakness and loss of breath on going upstairs.

Nitric-acid, loss of breath and palpitation, with anxiety on going upstairs.

Platina, pressure on genitals, aggravated by going upstairs.

Acetic-acid, loses breath on going upstairs.

Aurum-muriaticum, loss of breath (dyspnoea), and palpitation on going upstairs, or ascending a hill.

Cactus-grandifloris, palpitation of heart and sense of constriction of heart, worse on going upstairs. Also increase of dyspnoea from ascending stairs.

Stannum, faint sensation on going downstairs; can go up well enough.

Lobelia-inflata, dyspnoea worse from either ascending or descending.

